# Changes in regional heatwave characteristics as a function of increasing global temperature

**DOI:** 10.1038/s41598-017-12520-2

**Published:** 2017-09-25

**Authors:** S. E. Perkins-Kirkpatrick, P. B. Gibson

**Affiliations:** 1 Climate Change Research Centre, UNSW Australia, NSW, 2052 Australia; 2 ARC Centre of Excellence for Climate System Science, UNSW Australia, NSW, 2052 Australia

## Abstract

The Paris Agreement calls for global warming to be limited to 1.5–2 °C. For the first time, this study investigates how different regional heatwave characteristics (intensity, frequency and duration) are projected to change relative to increasing global warming thresholds. Increases in heatwave days between 4–34 extra days per season are projected per °C of global warming. Some tropical regions could experience up to 120 extra heatwave days/season if 5 °C is reached. Increases in heatwave intensity are generally 0.5–1.5 °C above a given global warming threshold, however are higher over the Mediterranean and Central Asian regions. Between warming thresholds of 1.5 °C and 2.5 °C, the return intervals of intense heatwaves reduce by 2–3 fold. Heatwave duration is projected to increase by 2–10 days/°C, with larger changes over lower latitudes. Analysis of two climate model ensembles indicate that variation in the rate of heatwave changes is dependent on physical differences between different climate models, however internal climate variability bears considerable influence on the expected range of regional heatwave changes per warming threshold. The results of this study reiterate the potential for disastrous consequences associated with regional heatwaves if global mean warming is not limited to 2 degrees.

## Introduction

Heatwaves, defined as prolonged periods of excessive heat^1^ are a distinctive type of extreme temperature that inflict disastrous impacts on human health^2–4^ infrastructure^5,6^, and biophysical systems^7,8^. Since as early as the 1950’s, increases in the in the duration, intensity and especially the frequency of heatwaves have been detected over many regions^9^. As anthropogenic influence on the global climate intensifies, future increases in heatwaves are unavoidable^10–15^. Some regions where intense heat is already common may become inhabitable ^16^, while tropical regions will experience extremely large increases in heatwave frequency due to low interannual variability^17,18^. Projected increases in heatwaves are dependent on the underpinning emissions scenario, with the largest changes anticipated under ‘business as usual’ (RCP8.5)^13–15,19^. The speeds at which heatwaves are changing are also more rapid under anthropogenic influence, with regional heatwave frequency trends commencing around 2010 or later unprecedented against a preindustrial climate^20^.

In December 2015, the United Nations Framework Convention on Climate Change (UNFCCC) held the 21^st^ Conference of the Parties, resulting in the Paris Agreement^21^. The first conventional aim of the agreement is to limit global warming by 2100 to “well below” 2 °C warmer than preindustrial conditions, with pursued efforts to limit warming to 1.5 °C^21^. Whilst universal targets are imperative for international agreement and measuring overall progress, they do not explicitly consider regional changes that may occur under specific warming thresholds^22–24^. Furthermore, it is unreasonable to assume that all regional climatological shifts will follow the global mean, including changes in corresponding extremes.

Larger increases in temperature extremes are expected respective to 2 °C mean global warming, however with considerable regional variation^24^. Global climate models project that annual minimum temperatures over the Arctic will reach 5.5 °C warmer than the regional preindustrial climate^24^, whereas annual maximum temperatures over much of the Northern Hemisphere, Central America and South Africa will be at least 3 °C warmer^24,25^. Also by 2 °C global warming, increases in annual maximum temperatures over 50% of land regions are expected to be almost 2 standard deviations (σ) warmer than pre-industrial conditions, with some tropical regions experiencing regular 3σ events^25^. A difference in global warming between 1.5° and 2 °C greatly increases the frequency of extreme temperatures over many regions^25,26^. Recent research also suggests that soil moisture-temperature feedbacks further amplify increases in warm extremes, in addition to the effect of increasing global temperature^27^.

While reported increases in regional extremes relative to global warming are concerning, it cannot be assumed that they are directly indicative of changes in heatwaves, since such studies have used simplified extreme temperature measures^24–26^. Heatwaves are a distinctive type of extreme temperature event, where anomalous conditions must occur over consecutive days. Thus, they can be considered via a number of characteristics (e.g. intensity, frequency, duration), as opposed to a single daily value that underpins annual maxima and minima events. According to many definitions, heatwaves are persistent exceedances of a given percentile, allowing for events to be relative to the regional climate^11,14,28^ and in some instances, the time of year^1,29^. However this also means that heatwave characteristics tend to display inter-annual variability given the dependence on a number of physical conditions^30–34^^.^ Moreover, the peak intensity of a heatwave is not necessarily the hottest day of a given year. Lastly, it is the sustained nature of heatwaves that impose more devastating impacts than extreme temperatures on a single day. Excessive human morbidity and mortality rates are clearly associated with sustained extreme temperatures^3,35^, as is substantial decreases in workplace productivity^36^, increased electricity demand coupled with decreased supply^6^, and potentially irreversible damage to vital ecosystems^7,37,38^. Since simplified measures of extreme temperature cannot deliver key information on heatwaves, an explicit investigation on how regional heatwave characteristics will change relative to global warming is warranted, however is currently lacking in the climate science literature. We anticipate that changes in heatwave frequency, intensity and duration relative to global warming will be highly regionally variable and will differ from prior work on more general measures of temperature extremes^24–26^.

For the first time, the present study investigates how different characteristics of regional heatwaves change relative to mean global warming. While there has understandably been substantial focus on universal thresholds of 1.5 °C and 2 °C, this study also considers heatwave changes at warmer thresholds, giving insight on the future landscape of heatwaves if Paris Agreement targets are not upheld. Two global climate model ensembles are employed, the Coupled Model Intercomparison Project Phase 5 (CMIP5)^19^; archive; and a 21-member version of Community Earth System Model (CESM)^39,40^. The former estimates projections across a suite of models of varying climate sensitivities, physical parameterizations and resolution, while the latter solely assesses the influence of internal variability (see Methods). Four heatwave characteristics are examined across a 5-month summer season, including the sum of heatwave days, the total number of discrete events, the length of the longest event and peak heatwave intensity^1^. Results are considered globally and for 21 land-based regions^41^, where both global mean warming and heatwave thresholds are relative to pre-industrial conditions. This combination of global mean temperature thresholds, climate models, and regional heatwave characteristics was purposely designed such that the findings of this study are immediately applicable to real-world heatwave adaptation and mitigation strategies that are centered on the agreed future global warming targets^21^.

## Results

### CMIP5 global median changes per °C warming

**Figure 1:**
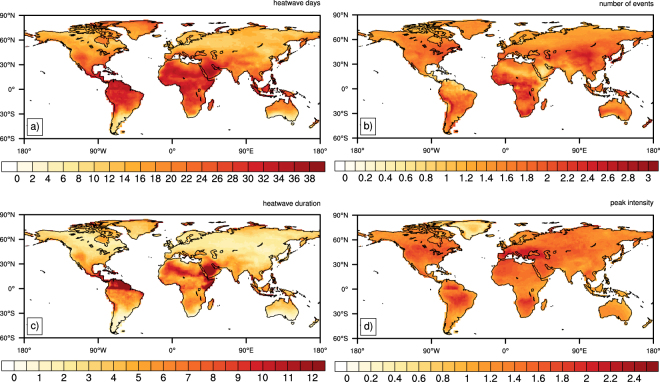
Median regression coefficients estimated from the CMIP5 model ensemble between global warming (°C) and seasonal (**a**) heatwave days; (**b**) number of events; (**c**) event duration; and (**d**) peak heatwave intensity. Created using NCAR Command Language (version 6.4.0) [Software]. (2017). http://dx.doi.org/10.5605/D6WD3XH5.

Figure 1 displays the CMIP5 ensemble median change in each heatwave characteristic per degree warming throughout the 21^st^ Century. Heatwave days (Fig. 1a) show the most striking changes in the tropics, with over 30 extra heatwave days per season over large parts of Africa, Central and South America and South East Asia, per °C of global temperature rise. This change is less severe in the mid to high latitudes, where 10–15 extra days are expected over Northern America, Europe and Russia. Over southern Australia and South America, a median of 4–8 extra heatwave days is expected for each degree of global warming.

Over most regions, the number of heatwaves per season (Fig. 1b) is projected to increase by approximately 1.5–2 events per degree of global warming. The exception is over central and southern Africa and central Asia, where a median increase of 2.5 events per season is projected. However, caution is recommended in interpreting changes in heatwave events per °C over some regions, as discussed further in Section 3.2.

The median change in the longest heatwave duration per season (Fig. 1c) is mostly between 1–3 days, with smaller increases at higher latitudes. Slightly larger increases of 4–6 days are projected per degree of global warming over India, southeast Asia, the United States and southern America. However, the longest event of the season is projected to increase by 10–12 days per degree of global warming across Central America, parts of Africa and the Middle East.

Over some regions, changes in heatwave amplitude (hottest heatwave day per season, Fig. 1d) are reasonably similar to increases in in global temperature. Over Australia and southeast Asia, heatwave amplitude is projected to increase approximately 1:1 with global temperature. For large parts of the world, the increase in heatwave amplitude is between 1.2–1.5 °C per degree of global warming, with values of up to 1.8 °C over the United States, parts of Africa and South America, and 2 °C over Europe.

### CMIP5 regional median changes per °C warming

**Figure 2:**
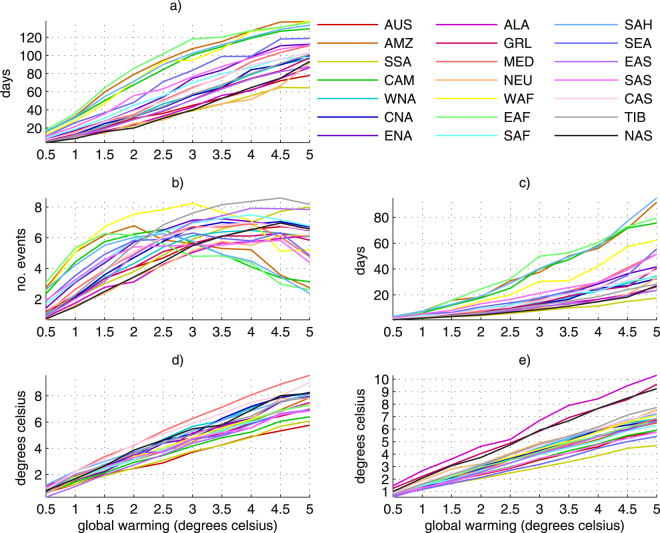
Regional (see Giorgi and Francisco, 2000) changes in heatwaves relative to 0.5 °C global warming thresholds estimated from the median of the CMIP5 model ensemble for (**a**) heatwave days; (**b**) number of events; (**c**) event duration; (**d**) peak intensity; and (**e**) regional mean warming. See table S.2 for region boundaries.

Consistent with Fig. 1, there is large regional variation in the median increase of heatwave characteristics (Fig. 2a–d) and regional mean warming (Fig. 2e) relative to global temperature increase. Over high latitude areas (ALA, GRL, NAS; see table S.2 in the supplemental material), regional warming (Fig. 2e) is almost double global warming, whereas an approximately 1:1 increase occurs over lower latitude regions (SSA, SEA, SAS, AUS). Figure 2e may be used in conjunction with heatwave changes in Fig. 2a–d to greater understand differences in regional changes relative to universal temperature increases.

Regional changes in heatwave days (Fig. 2a) are mostly linear, with a large spread. Fig. 2a also supports Fig. 1a, where more rapid increases in heatwave days relative to global temperature occur over tropical regions compared to those at higher latitudes. By 5 °C global warming, the number of heatwave days per season could increase by between 60 days (SSA) to 120 days (EAF, AMZ, WAF), suggesting that heatwave conditions will become the new normal if 5 °C global warming is reached. From 1 °C warming, a regional divergence is evident, where lower latitude regions start to display faster increases in heatwave days. Note that regions with larger overall increases display slower rates of change above 2.5–3 °C, as the maximum number of seasonal heatwave days (~150) is approached. These results indicate that the threshold of global warming reached manifests differently in terms of regional changes in heatwave days.

It is clear that regional changes in the number of seasonal heatwaves and global temperature increase are not linearly associated (Fig. 2b). Indeed, there is a decrease in events for some lower latitude regions at 1.5–2 °C (EAF, AMZ) and 3 °C warming (WAF, SAH), resulting in a negative median coefficient for some regions (Table 1). For most other regions, the rate of increase in heatwave events slows at around 3 °C global warming, with some regions demonstrating a decline from 4.5 °C (SEA, SAS, MED). When considered in conjunction with Fig. 2a, it is likely that large increases in heatwave days are resulting in long, continuous events without any reprieve. At 1.5 °C warming, the number of events can increase by 2 (NAS, NEU) to over 6 extra events per season (WAF, AMZ, EAF). Such results indicate the high sensitivity of heatwave frequency to the total amount of global warming reached.

**Table 1:** Regional coefficients of heatwave changes per °C global warming estimated from the CMIP5 ensemble. The first figure in each cell is the ensemble median, followed the range (99^th^–1^st^ percentiles).

Region	Heatwave days	Number of events	Length of longest event	Peak intensity
AUS	16.3 (9.6–21.7)	1.3 (0.8–1.9)	4.6 (2.1–9.5)	1.2 (0.8–1.4)
AMZ	26.6 (20.9–37)	0 (−1.5–2.9)	16.2 (5.3–31.4)	1.5 (1.1–2)
SSA	14.8 (6.2–21.3)	1.7 (0.6–2.4)	3.4 (1.6–6.7)	1.2 (0.5–1.7)
CAM	27.3 (13.5–34.9)	−0.1 (−1.7–1.6)	17.5 (5.7–29.8)	1.3 (0.6–1.8)
WNA	20.9 (13.3–30.7)	1.4 (0.5–2.2)	6.4 (2.7–13.9)	1.7 (1.1–2.1)
CAN	20.1 (8.4–32.9)	1.5 (0.1–2.5)	6.1 (1.6–15.7)	1.7 (0.9–2.6)
ENA	26.3 (16.6–36.2)	1.4 (−0.7–2.9)	8.4 (2.5–26.7)	1.6 (0.9–2.4)
ALA	16.9 (10.4–27.3)	1.4 (−0.1–2.0)	4.5 (2.6–15.1)	1.4 (0.6–3)
GRL	19.8 (14.8–20.2)	1.4 (−0.4–2.4)	6.1 (3.3–23)	1.3 (0.7–2.2)
MED	24.2 (17.4–31.3)	1.5 (0.4–2.2)	8.5 (4–17.5)	1.9 (1.6–2.4)
NEU	16.1 (7.4–25.1)	1.3 (0.3–2.2)	4.2 (1.9–13)	1.5 (0.6–2.4)
WAF	26.4 (7.5–37.9)	0.8 (−2.4–3)	13.3 (2–32.1)	1.5 (1.2–1.8)
EAF	25.6 (8.6–37.2)	−0.3 (−2.4–1.8)	16.9 (2.5–34.5)	1.5 (1.1–2.5)
SAF	22.6 (14.8–21.1)	1.5 (0.3–2.3)	6.6 (2.8–13)	1.5 (1.2–2.5)
SAH	28.2 (22–32.8)	0.3 (−1.4–1.9)	17.5 (6.9–30)	1.6 (1.3–1.8
SEA	25.9 (11.9–35)	1.2 (−2–2)	7.8 (2.7–28.8)	1.4 (0.5–2)
EAS	20.2 (12.4–29.8)	2 (0.6–2.8)	4.8 (2.7–28.8)	1.5 (1.2–2.2)
SAS	23.4 (12.3–29.5)	1 (−1–1.9)	7.8 (3.9–19.8)	1.3 (1–1.7)
CAS	23.1 (16.5–30.2)	1.5 (0.4–2.2)	6.8 (3.6–16.7)	1.7 (1.4–2.4)
TIB	22.7 (17.3–29.7)	2.2 (0.7–3.6)	5.5 (3.4–13.9)	1.6 (1.3–2.2)
NAS	17 (7.8–24.8)	1.52 (0.4–2.3)	4.5 (1.4–9.9)	1.8 (0.5–2.6)

The duration of the longest event (Fig. 2c) is most rapid over tropical regions (SAH, AMZ, CAM, EAF), similar to Fig. 1b. Note that if 5 °C of global warming is reached, heatwaves in these regions could last for over 80 days. However, if warming were limited to 2 or 3 °C, they would be substantially shorter at 20, and 40–50 days, respectively. Most other regions see a sharper increase from 3.5 °C, suggesting that the length of heatwaves are more sensitive to higher increases in global temperature in these regions. Over these regions, a global warming limit of 2.5 °C could result in heatwaves that are an extra 5–20 days in length, relative to pre-industrial times.

Figure 2d displays a highly linear increase in regional median peak heatwave intensity relative to global warming. Consistent with previous studies^26^ the largest increase occurs over the Mediterranean (MED), where heatwave intensity could be 9 °C hotter in a 5 °C world, relative to a pre-industrial climate. In a 2.5 °C world, heatwaves could be an extra 2.5 °C (AUS, SSA) to 5 °C (MED, CAS) warmer. This is a notable increase compared to a world at 1.5 °C, where heatwaves are approximately 2 °C (EAS, SSA) to 3 °C (MED, CAS) warmer.

Regional median return intervals of intense heatwaves projected by CMIP5 also diminish at a non-linear rate per global warming threshold (Table 2). By 4 °C global warming or earlier, almost all regions experience an intense heatwave yearly that occurred only once every 30 years between 1861–1890. Across all regions, there is a large difference in return intervals between 1.5 °C and 2.5 °C global warming. In some cases, an intense heatwave occurs twice as often at 2.5 °C (e.g. GRL, WNA, AUS), and over other regions this increase in frequency between 1.5 °C and 2.5 °C is nearer to 3-fold (e.g. TIB, ENA). Lower thresholds of global warming will therefore mean that the occurrences of extremely intense events are kept to a minimum.

**Table 2:** CMIP5 ensemble median change in frequency of a 1-in-30 year peak heatwave intensity that originally occurred during 1861–1890 per 0.5 °C global warming. A value of 10 means an event of the same intensity occurs once every 10 years, on average, at the specific global warming threshold.

	0.5	1	1.5	2	2.5	3	3.5	4	4.5	5
AUS	10.00	5.00	2.73	1.88	1.30	1.15	1.03	1.02	1.00	1.00
AMZ	7.50	4.00	1.88	1.25	1.07	1.00	1.00	1.00	1.00	1.00
SSA	10.00	4.29	2.50	1.71	1.33	1.11	1.03	1.00	1.00	1.00
CAM	15.00	7.50	2.73	1.43	1.11	1.00	1.00	1.00	1.00	1.00
WNA	20.00	7.50	2.31	1.40	1.15	1.07	1.00	1.00	1.00	1.00
CAN	15.00	10.00	4.29	2.50	1.58	1.25	1.11	1.03	1.03	1.00
ENA	10.00	6.00	3.75	1.88	1.36	1.07	1.00	1.00	1.00	1.00
ALA	15.00	15.00	7.50	4.62	3.33	2.50	1.76	1.40	1.15	1.11
GRL	15.00	6.00	2.73	1.67	1.36	1.11	1.03	1.00	1.00	1.00
MED	12.00	3.75	1.76	1.25	1.03	1.00	1.00	1.00	1.00	1.00
NEU	20.00	15.00	5.00	3.16	2.31	1.67	1.25	1.11	1.00	1.00
WAF	6.00	2.73	1.58	1.11	1.00	1.00	1.00	1.00	1.00	1.00
EAF	7.50	2.31	1.15	1.03	1.00	1.00	1.00	1.00	1.00	1.00
SAF	12.00	3.75	2.07	1.30	1.11	1.03	1.00	1.00	1.00	1.00
SAH	5.00	2.00	1.15	1.00	1.00	1.00	1.00	1.00	1.00	1.00
SEA	12.00	6.67	4.29	2.73	2.22	1.71	1.43	1.20	1.05	1.02
EAS	15.00	15.00	4.29	1.76	1.36	1.03	1.00	1.00	1.00	1.00
SAS	6.00	5.00	2.86	1.67	1.20	1.03	1.00	1.00	1.00	1.00
CAS	10.00	4.29	1.94	1.25	1.07	1.00	1.00	1.00	1.00	1.00
TIB	15.00	6.67	3.75	1.67	1.20	1.03	1.00	1.00	1.00	1.00
NAS	8.57	7.50	3.00	1.82	1.25	1.07	1.00	1.00	1.00	1.00

### Variability in heatwave changes

**Figure 3:**
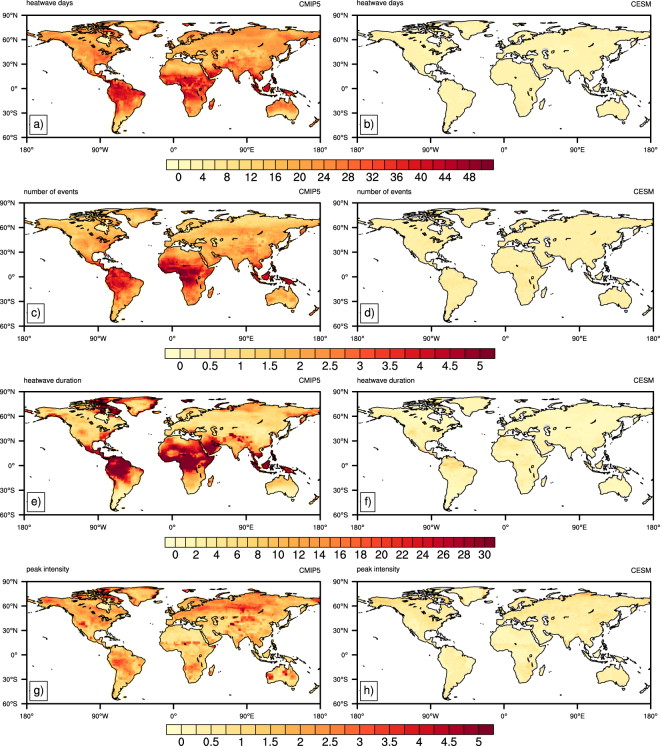
Spread in the regression coefficient estimated from CMIP5 models (left column) and the CESM ensemble (right column) for (**a**,**b**) heatwave days; (**c,d**) number of events; (**e,f**) event duration; and (**g,h**) peak intensity. The spread is given by the ensemble 1^st^ percentile subtracted from the ensemble 99^th^ percentile. Created using NCAR Command Language (version 6.4.0) [Software]. (2017). http://dx.doi.org/10.5605/D6WD3XH5.

The results above are based on CMIP5 medians. However, variability exists among ensemble members, suggesting that the relationship between regional heatwave characteristics and global warming is model dependent to some extent (Fig. 3, Table 1). Note that with the exception of peak intensity (Fig. 3f, Table 1) tropical regions display the greatest differences between models, which are also regions most sensitive to global temperature increases (Figs 1 and 2). The overall spread in changes of heatwave days per °C among the CMIP5 models may be as large as 40 (Fig. 3a), while the overall number of events and the duration of the longest event may differ by up to 4 events (Fig. 3c) and 30 days (Fig. 3e), respectively. This means that for a given threshold of global warming, the difference in projections between two climate models may be up to 40 heatwave days per season and 4 discrete events, respectively. Note that the spread is markedly reduced outside tropical regions, where heatwave days, event number and longest duration vary by 8–20 days, 1–2.5 events, and 4–12 days, respectively (Fig. 3, Table 1). This indicates higher model agreement on how sensitive heatwave changes are to global temperature increases over these areas. Ensemble spread in peak heatwave intensity shows no regional association, generally being between 1.5–2.5 °C (Fig. 3g; Table 3).

**Table 3:** Median estimate of warming per °C global warming due to internal variability. (CESM(99^th^–1^st^)/CMIP5 (99^th^–1^st^)).

Region	Heatwave days	Number of events	Length of longest event	Peak intensity
AUS	36	50	34	47
AMZ	28	21	17	35
SSA	34	61	27	48
CAM	22	37	23	30
WNA	41	55	37	46
CAN	53	78	44	68
ENA	43	46	27	51
ALA	39	70	25	90
GRL	30	50	16	50
MED	54	61	35	67
NEU	46	59	33	54
WAF	25	32	12	28
EAF	30	43	12	37
SAF	36	37	40	66
SAH	39	26	15	36
SEA	25	28	12	51
EAS	41	48	26	52
SAS	26	31	17	67
CAS	43	59	21	52
TIB	38	50	22	46
NAS	25	39	20	32

Figure 3b,d,f and h suggest that the influence from internal climate variability is relatively low. For all heatwave characteristics, the coefficient per °C varies little regionally, and is fractional compared to the respective spread of the CMIP5 ensemble. A spread of no more than 4 days (Fig. 3b), 0.5 events (Fig. 3d), 3 days (Fig. 3f), and 0.5 °C (Fig. 3h) can be expected per °C of global warming for the number of heatwave days, total number of events, the length of the longest event and peak intensity, respectively, due to the internal variability of the climate system. Thus, any variation in the rate of heatwave changes is largely dependent on physical differences between climate models (e.g. processes resolved, parameterization schemes, resolution, overall climate sensitivity).

**Figure 4:**
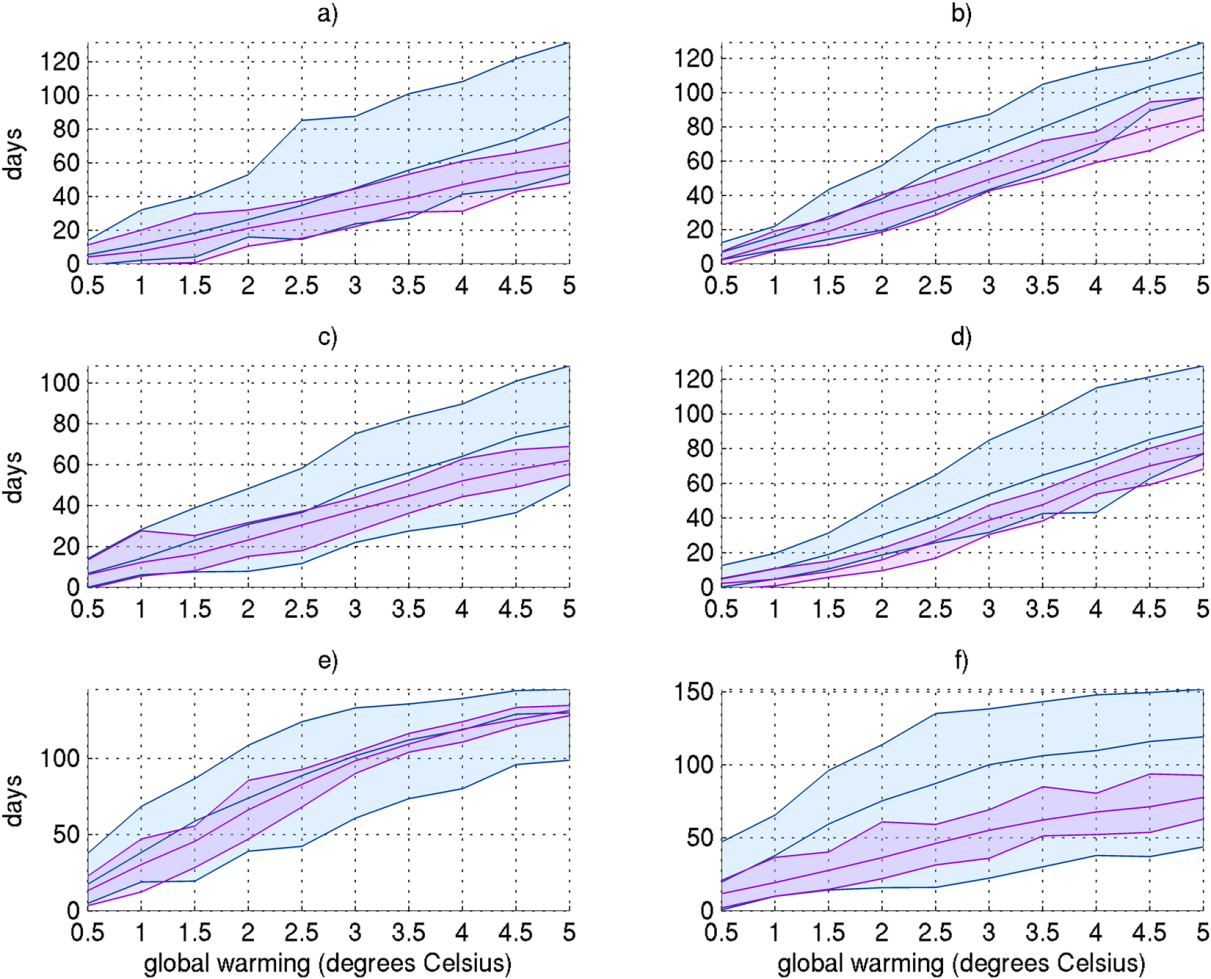
Ensemble spread (99^th^ percentile – 1^st^ percentile) of increases in the number of heatwave projected by the CMIP5 ensemble (blue) and CESM ensemble (purple) per 0.5 °C global warming, for (**a**) Alaska; (**b**) the Mediterranean; (**c**) Australia; (**d**) East Asia; (**e**) the Amazon, and (**f**) East Africa. These regions were chosen as they are representative of all 21 regions analysed. Note that the spread due to internal variability (estimated from CESM) is reasonably consistent across the thresholds. See table S.2 for region boundaries.

However, internal variability plays a larger role on the overall changes in regional heatwaves at a given global warming threshold. Over many regions, this role does not diminish as anthropogenic influence on the global climate increases (Fig. 4. and Table 3). In terms of regional increases in the number of heatwave days, internal climate variability may account for ~25–50% of the projected spread when each ½ °C threshold is reached (Table 3, Fig. 4). For the number of events, the duration of the longest event and peak intensity, internal climate variability may respectively account for 21–70%, 12%–35%, and 28%–67% of the projected variation, depending on the region. In general, this influence is larger over higher latitude regions (e.g. ALA, CAN, NEU) than those in the tropics (e.g. AMZ, SEA, WAF). Moreover, influence of internal variability is consistent through time over most regions (Fig. 4), with the exception of the tropics (AMZ), where the influence of internal variability diminishes as global temperature increases. Thus, while the average rate of change in heatwaves relative to global warming is largely dependent on the physical representation of the climate system, internal climate variability should also be taken into account when determining the overall regional change projected per specific global warming threshold.

## Discussion and conclusion

The present study expands upon existing literature investigating how global temperature increases correspond to changes in regional temperature extremes^24–26^. A novel aspect of this study involves separately investigating changes in multiple heatwave characteristics (intensity, frequency and duration) relative to global warming – until now, we have not known how regional heatwaves will change when particular global warming thresholds are reached. This is an essential addition to the literature because the vast array of adverse impacts caused by heatwaves^2,5,35,36^ are due to their distinctive characteristics. As such, it cannot be assumed that changes in more simplistic measures of extreme temperature directly correlate to changes in heatwaves. While the discussed relationships between increasing global temperature and heatwave intensity and duration are respectively comparable to increases in annual maximum temperature^24^ and warm spells^25^, they are not identical. Moreover, for the first time, this study has established the alarmingly fast rate of increase in heatwave days over many regions relative to global warming, as well as the transition to a constant heatwave state over tropical regions within the bounds of the Paris Agreement. Our results highlight the clear advantages of limiting global warming to 1.5 °C^26^, specific to changes in regional heatwaves. Additionally, we have provided new insight into how heatwaves will change relative to warming thresholds beyond 2 °C, which has not been previously established in the scientific literature. These are essential to consider so that we are prepared for such conditions if the 1.5° and 2 °C benchmarks are exceeded. Indeed, knowing such increases beyond 2 °C strengthens the importance of ensuring the success of the Paris Agreement. Future work could involve investigating the role of the land surface in amplifying regional heatwaves, in addition to links with global temperature rise^27,42^.

By employing the multi-model CMIP5 archive, we have demonstrated the varying rates at which regional heatwave characteristics increase relative to a universal temperature trajectory. While most heatwave changes are linear with respect to global warming (Fig. 2), the main exception is the number of discrete events per season. From 2 °C global warming onwards and particularly over tropical regions, the regional number of events may decrease. While this result is consistent with studies demonstrating the relatively rapid climatological shift of the tropics^17,43,44^, it potentially imposes adverse regional impacts, as a perpetual heatwave state will emerge each summer once 2 °C global warming is reached and exceeded. Indeed, impacts on local ecosystems could be devastating, as tropical climates will be pushed into uncharted territories. Regional changes in heatwave days are also quite striking. Increases in heatwave days may exceed 30 days per °C of global warming over tropical and arid regions. Changes of around 20 days per °C are anticipated over northern high latitudes, and 10–20 days per °C over southern high latitudes (Fig. 1a). Thus, if global warming is not capped at 2 °C or less, regional increases in heatwave days may span 60–120 *extra* days per summer by the end of this century, relative to a pre-industrial climate (Fig. 2). Generally speaking, the peak intensity of heatwaves scales reasonably close to the amount of global warming (Fig. 1d), with regional variations of 1–1.8 °C, per °C of global warming. The Mediterranean and Eurasian regions demonstrate the largest change, where peak heatwave intensity may exceed 8 °C by 2100 if global warming is not constrained. However, it is worth remembering that the occurrence of extremely intense heatwaves increases far more rapidly. By 1.5 °C global warming, almost all regions experience heatwaves every 4 years that occurred every 30 years when anthropogenic influence on the climate was negligible (Table 2).

While median projections from the CMIP5 ensemble are useful in approximating heatwave changes per global warming threshold, utilizing the multi-member CESM ensemble has shed light on the influence internal climate variability. Our results suggest that the overall rate at which all heatwave characteristics change per °C warming is largely independent from internal variability (Fig. 3b,d,f and h). This means that despite what sequence of internal variability actually occurs, the overall speed of heatwave changes relative to global warming will be extremely similar. As such, it is the overall physical representation of the climate system, as governed by the choice of model, that will largely dictate the rate of change in heatwaves per °C of global warming (Fig. 3a,c,e and f; Table 1). This is amplified over tropical regions for heatwave frequency and duration, where overall projected changes in heatwaves are also the greatest^17,44^ (Figs 1 and 2). Even over higher latitudes, the rate of change per °C global warming in heatwave days, number, duration and peak intensity may vary by as much as 24 days; 2–2.5 discrete events; 10 days; and 1.5–3 °C respectively, dependent on the choice of climate model/s used.

However, there is considerable influence from internal variability when projecting changes in heatwaves per individual global warming threshold, as opposed to the overall relationship (Fig. 4; Table 3). Over some regions and for some heatwave characteristics, internal variability accounts for at least 50% of the range of expected heatwave changes per ½ °C global warming, though in most cases explains between 20–30% of the range (Table 3). This is an important point, since even if our understanding of the response of the climate system to anthropogenic forcing was perfected (i.e., we created a “flawless” physical climate model), a range of changes per global warming threshold should still be anticipated due to the influence of internal climate variability which remains unpredictable. Therefore, at the very least, fluctuations of the order of those presented in Table 3 should be employed in the construction of adaptation and mitigation policies regarding heatwave changes relative to specific global warming thresholds.

This is relevant to model evaluation also, and implies that median ensemble projections presented in the present and other similar studies^24,25^ should not be deemed incorrect if the true change is within the regional range of internal variability. It is worth highlighting the important difference between the influences of variability per universal threshold, and on the overall trajectory of heatwaves discussed above. The latter assumes a particular course variability will take, as sampled by the 21 ensemble CESM members, where little influence on the rate of heatwave changes is measured across the sample. However, we do not know what the future course of climate variability will be, and a similar overall trend can result in different absolute changes at specific universal temperatures. Moreover, due to computational limitations, the analysis of internal variability in this study is limited to one physical model (CESM). It is plausible that other climate models, should they provide an appropriate ensemble, will differ in their estimated influence of internal variability on heatwave changes relative to global warming^20^. However, the use of the CESM ensemble against CMIP5 clearly demonstrates that the structural and physical differences across climate models, and not internal variability, largely accounts for the variation in the scaling of heatwaves against global warming. While we believe a qualitatively similar result would be gained from a using a different climate model with a multi-member ensemble, future work could endeavor to test this hypothesis.

It is also critical to highlight the substantial differences in heatwave changes, dependent on the overall amount of global warming reached, and what this may infer for impacts. For example, anywhere between 3 to 20 extra heatwave days will be expected on average between global warming thresholds of 1.5 °C and 2 °C, where peak heatwave intensity will warm by approximately 0.5 °C, depending on the region (Fig. 2). While changes in most regional heatwave characteristics are predominantly linear relative to global warming, this does not infer that changes in the *impacts* of heatwaves will also be linear^35^. For example, Australian fruit bats perish at specific temperature thresholds^7^. Increases in peak heatwave intensity beyond these thresholds may see this species (and others) wiped out entirely, particularly when combined with perpetual heatwave conditions. Similarly, public infrastructure may be far more prone to failure as perpetual heatwaves become the new normal^5^. Current health impacts of heatwaves on humans generally affect the elderly and chronically ill^4^. Further combined increases in event intensity, frequency and duration associated with higher global warming thresholds will likely see a larger proportion of the population at risk^35,45,46^, having knock-on effects to public health resources. Adding additional complexity, the timeframe of these potentially catastrophic impacts will be highly regional, dependent on local heatwave changes relative to the total amount of global warming reached, as well as the underlying vulnerability of the local population^35,36^. Thus, many more challenges in defending against the impacts of heatwaves may be expected per 0.5 °C of global warming beyond the Paris agreement. Although some are already underway^35^, a large range of targeted, impact-based studies is essential in understanding what exactly these challenges will be, as well as the implications and overall cost of heatwave impacts if targets outlined in the Paris Agreement are not met.

It is important to note that some degree of uncertainty remains in terms of the global climate response to increased anthropogenic influence, and in terms of how regional heatwaves will change. The transient climate response of the CMIP5 models (i.e. the response of global temperature to a doubling of atmospheric carbon dioxide at 1% increase per year over 70 years) varies between 1.2 °C and 2.4 °C^47^, with the observation-based response still debated but considered generally consistent with CMIP5^47,48^. While recent years has seen an increase in research by the global climate community in defining the physical mechanisms of heatwaves^31,32,49,50^, it is challenging for climate models to simulate these processes both currently as well as their changes in the future^51^. Moreover, the resolution of global climate models is likely too coarse to fully simulate such processes (e.g. synoptic systems and land surface interactions) and their intricate connections, rendering projections of heatwaves general approximations. All these factors and more undoubtedly contribute to the spread in the pace of regional heatwave changes and their relationships to global temperature increases among the CMIP5 ensemble, which we have shown here. Thus, it is advisable that a single, or small group of models is *not* employed to comprehensively explore the implications of global temperature change on regional heatwaves for impacts purposes, as it cannot be guaranteed that key, underpinning physical processes and physical responses will be adequately represented by such a sample. While a bigger model ensemble introduces a larger range of responses (Figs 3 and 4), it is imperative this is accounted for when examining the influence specific global warming thresholds - it is extremely difficult, if not impossible, to know what the true response of the climate system to such targets will be.

Attention should also be drawn to the plethora of heatwave definitions that exist in both climate and impacts literature. There is no universal heatwave metric, nor will one likely ever exist, owing to the multiple physical characteristics^30–34,49^ heatwaves have and their vast array of impacts^4,6,7,16,35–38,45,46^. Indeed, many impacts-based fields have their own specific definitions, directly relating to the impact at hand^1^. This study employed the percentile-based definition against a baseline climate (see Methods) for multiple reasons – it is practical for different climates; it has been successfully used to derive changes in heatwaves from different types of climate data; and multiple heatwave characteristics can be derived for a range of such impacts^1^. However, all percentile-based definitions show very large changes in heatwave frequency and duration over tropical regions, due to the small temperature distributions of these areas. As a consequence of such increases, some studies suggest serious impacts to human health and productivity, especially if coupled with a rise in humidity^36,45^. Moreover, Tropical ecosystems successfully function within a tight temperature range – small, yet regular deviations above this could result in disastrous ramifications. However there is and always will be a place for absolute-based heatwave definitions that do not require a baseline climate, for example to understand how particular species may cope with changes in frequency of certain future temperatures^7^ or potential changes of particular diseases that are directly correlated with certain temperature thresholds^4^.

In summary, for the first time, the present study has demonstrated the varying and concerning rates regional heatwave intensity, frequency and duration are projected to change, relative to global warming. While universal targets, such as those outlined in the Paris Agreement, are essential for global action on climate change, they present very different trajectories at the regional scale. It is imperative that such trajectories are well understood, inclusive of uncertainties due to internal climate variability and the overall response of the climate system to increased anthropogenic forcing, so that effective, long-term adaptation and mitigation heatwave policies are well-informed. Moreover, to avoid considerable changes to the nature of regional heatwaves it is absolutely crucial that global warming is minimized within the bounds of the Paris agreement. This study is the first to explicitly analyse how different characteristics of regional heatwaves will change relative to global warming beyond 2 °C, which will likely infer devastating impacts if anthropogenic climate change is not constrained as soon as possible.

## Methods

### Data

The bulk of analysis employs heatwave projections from the CMIP5 model archive^19,52^. Participating models required daily data between 1861–2005 for the historical experiment, and from 2006–2100 for RCP8.5, resulting in 27 models (see Table S.1 in the supplemental material). This experiment was chosen based on the number of models available, and evidence suggesting that it is our current emissions trajectory^53^. To avoid biasing results towards one or a handful of models, only the first realization of each model was used. Identical analyses were performed using the same models for the RCP4.5 experiment, however results were very similar to those reported in this study and were omitted for the sake of brevity. Since RCP4.5 is considered a “middle of the road” scenario^52^, global warming does not exceed 3.5 °C, and therefore regional changes in heatwaves are significantly less. This is in agreement with recent work^26^, where little difference is found between the relationship of extremes and global warming between different emissions scenarios, relative to a large overall difference by the end of the 21^st^ century. Fig. S.1 in the supplemental material demonstrates the temporal range of each 0.5 °C threshold under both experiments, which are generally reached later in time under RCP4.5. Thus, while relationships between global average temperature and heatwaves are very similar across the experiments, the impacts on heatwaves will also be felt later if RCP4.5 became our future trajectory.

In order to investigate the influence of internal variability on the relationship between global warming and heatwaves, we employ a 21-member ensemble of a global climate model (Community Earth System Model; CESM). Specifically, version 1.0.4 was employed, which includes the Community Atmosphere Model version 4 at 1.875° × 2.5° global resolution^39,40^. All ensemble members are driven by identical external forcings. From 1950–2005 all members are forced with historical anthropogenic greenhouse gas and aerosol concentrations, and natural forcings. From 2006–2100 prescribed RCP8.5 forcings are employed. Each member only differs in their initial conditions, where on the 1^st^ of January 1950 random perturbations on the order of 10^−13^ are imposed on atmospheric temperature^40^. Despite this minute alteration, a substantial amount of variability is induced across the ensemble providing an ideal platform for this study. We exclude the first 5 years of each historical simulation for spin-up. Since CESM’s historical simulation commences in 1950, we employ the 982-year control run as a proxy for the earlier historical period, as discussed below.

### Calculating heatwaves

Before heatwaves were calculated, all model realizations were fitted with a land-sea mask. We employ the maximum temperature (T_max_) heatwave definition ^1^. In summary, daily T_max_ must exceed the calendar-day 90^th^ percentile for at least three consecutive days for a heatwave to be declared. The 90^th^ percentile is calculated from a smoothed 15-day moving average, such that it is relative to the time of year as well as the location. We consider heatwaves occurring during an extended summer, spanning November-March in the Southern Hemisphere and May-September in the Northern Hemisphere. All heatwaves are calculated at the grid box level. To determine how heatwaves have changed relative to a climate under little anthropogenic influence, percentile periods span 1861–1890 in the CMIP5 models, and a random 30-year period selected from the control run for CESM. Note there no detectable differences in percentiles from 500 30-year periods in the CESM control^20^.

Once heatwaves are identified, four characteristics^1,11^ are computed for each season, individually for each model simulation. These include:

- The total number of days that a part of a heatwave at least 3 days long;
- The duration of the longest event;
- The number of discrete heatwaves, and
- The peak intensity (the hottest day of the hottest event).

Note that the peak intensity does not always align with the hottest annual day (i.e. TXx)^24^, since the hottest event is first calculated by the largest average of each discrete event, and the hottest heatwave day (i.e. the peak intensity) is then extracted from this event. It is plausible for the hottest annual day to either fall within an overall cooler heatwave event, or as part of hot weather lasting less than 3 days. This results in an annual value per characteristic for both experiments at each land-based grid box. Regional averages for each characteristic are computed for all “Giorgi” regions^41^, described in Table S.2 of the supplemental material. Regional analysis also considers the change in return period for a 1-in-30-year peak intensity event relative to a pre-industrial climate; and changes in regional temperature, relative to global temperature (see below). Global maps for CMIP5 models (Figs 1 and 3) were compiled after re-gridding heatwave characteristics of each model to 1° × 1°, while CESM global analysis (Fig. 3) remained at the models’ native resolution.

### Comparing to global mean temperature

Using monthly data, annual area-weighted mean global temperature was calculated. Similar to heatwaves, anomalies in mean global temperature are calculated relative to 1861–1890 for the CMIP5 models and a random 30-year control period for CESM. This allows for an analysis on how global temperature has increased relative to the preindustrial world^21^. Anomalies of 0.5 °C increments up to 5 °C were obtained, where a specific threshold is ‘reached’ when it occurs for at least 5 consecutive years. To investigate changes relative to each global warming threshold, each heatwave characteristic is extracted for the same 5-year period and averaged.

Ordinary least squares regression coefficients, quantifying the relationship between global temperature increase and heatwaves, were calculated at the grid box level between the sustained 0.5 °C warming increments and each heatwave characteristic. We present the coefficients relative to 1 °C increases in global average temperature. The main exception is to the number of events, specifically over tropical regions, as discussed in section 2.1. Regression Coefficients were individually calculated per simulation, from which the ensemble median and spread were calculated. The CMIP5 ensemble median is presented in Fig. 1 and Table 1. Ensemble spread, defined as the difference between the 99^th^ and 1^st^ percentile, are presented for both CMIP5 and CESM in Fig. 2, and regionally for CMIP5 in Table 1.

We also compute the regional change in frequency of very intense heatwaves. The hottest peak intensity event between 1861–1890 is extracted on a regional basis. Return intervals, as presented in Table 2, are then recalculated relative to each region once each 0.5 °C global warming threshold is sustained, as described above.

Lastly, we also present the absolute change in each heatwave characteristic, computed for each region in Table S.2 by taking the relative median of the CMIP5 ensemble at each sustained 0.5 °C threshold (Fig. 2). While the ensemble spread of absolute changes cannot be detailed due to article length restrictions, Fig. 4 presents the ensemble spread in absolute changes of heatwave days for CMIP5 and CESM over six regions. These regions were selected since they cover key climates, and are good representatives of results over other regions. Also at the regional level, we present the proportion of absolute changes in each characteristic due to internal variability. This is computed by:

1 $${\rm{P}}=100\ast ({\rm{CESM}}({99}^{{\rm{th}}}-{1}^{{\rm{st}}})/{\rm{CMIP}}5({99}^{{\rm{th}}}-{1}^{{\rm{st}}}))$$

where CESM and CMIP5 refer to the relative ensemble and P is the proportion expressed as a percentage. Note that P is calculated per 0.5 °C threshold, however is expressed as the median across all thresholds in Table 2.

## Electronic supplementary material

supplementary material

## References

[1] Perkins SE, Alexander LV (2013). On the measurement of heat waves. J. Climate.

[2] McMichael AJ, Lindgren E (2011). Climate change: present and future risks to health, and necessary responses. J. Intern. Med..

[3] Coumou D, Rahmstorf S (2012). A decade of weather extremes. Nat. Clim. Change.

[4] Loughnan, M. E., A spatial vulnerability analysis of urban populations during extreme heat events in Australian capital cities 2013.

[5] Miller, S., Muir-Wood, R. & Boissonnade, A. An exploration of trends in normalized weather-related catastrophe loss, in H. Diaz and R. Murnane (eds) *Climate extremes and society*, Cambridge: Cambridge University Press, 225-247 (2008).

[6] McEvoy D, Ahmed I, Mullett J (2012). The impact of the 2009 heat wave on Melbourne’s critical infrastructure. Loc. Environ..

[7] Welbergen JA, Klose SM, Markus N, Eby P (2008). Climate change and the effects of temperature extremes on Australian flying-foxes. Proc. Roy. Soc. Lon. B: Biol. Sci..

[8] Karoly DJ (2009). The recent bushfires and extreme heat wave in southeast Australia. Bull. Aust. Met. Ocean. Soc..

[9] Perkins SE, Alexander LV, Nairn JR (2012). Increasing frequency, intensity and duration of observed global heatwaves and warm spells. Geophys. Res. Lett.

[10] Meehl GA, Tebaldi C (2004). More intense, more frequent, and longer lasting heat waves in the 21st century. Science.

[11] Fischer EM, Schär C (2010). Consistent geographical patterns of changes in high-impact European heatwaves. Nat. Geosci..

[12] Diffenbaugh NS, Ashfaq M (2010). Intensification of hot extremes in the United States. Geophys. Res. Lett..

[13] Cowan T (2014). More frequent, longer, and hotter heat waves for Australia in the twenty-first century. J. Climate.

[14] Russo S (2014). Magnitude of extreme heat waves in present climate and their projection in a warming world. J. Geophys. Res.: Atmospheres.

[15] Schoetter R, Cattiaux J, Douville H (2015). Changes of western European heat wave characteristics projected by the CMIP5 ensemble. Clim. Dyn..

[16] Pal JS, Eltahir EA (2016). Future temperature in southwest Asia projected to exceed a threshold for human adaptability. Nat. Clim. Change.

[17] Perkins SE (2011). Biases and model agreement in projections of climate extremes over the tropical Pacific. Earth Int..

[18] Herold N, Alexander L, Green D, Donat M (2017). Greater increases in temperature extremes in low versus high income countries. Environ. Res. Lett..

[19] Taylor KE, Stouffer RJ, Meehl GA (2012). An overview of CMIP5 and the experiment design. Bull. Amer. Meteorol. Soc..

[20] Perkins-Kirkpatrick SE, Fischer EM, Angélil O, Gibson PB (2017). The influence of internal climate variability on heatwave frequency trends. Environ. Res. Lett..

[21] UNFCCC. *Adoption of the Paris Agreement*. Report No. FCCC/CP/2015/L.9/Rev.1, http://unfccc.int/resource/docs/2015/cop21/eng/l09r01.pdf (UNFCCC, 2015)

[22] Lehner F, Stocker TF (2015). From local perception to global perspective. Nat. Clim. Change.

[23] Sutton RT, Dong B, Gregory JM (2007). Land/sea warming ratio in response to climate change: IPCC AR4 model results and comparison with observations. Geophys. Res. Lett..

[24] Seneviratne SI, Donat MG, Pitman AJ, Knutti R, Wilby RL (2016). Allowable CO2 emissions based on regional and impact-related climate targets. Nature.

[25] Schleussner C-F (2015). Differential climate impacts for policy-relevant limits to global warming: the case of 1.5-°C and 2-°C. Earth Sys. Dyn. Discuss..

[26] King AD, Karoly DJ, Henley BJ (2017). Climate extremes and Population exposure at 1.5 and 2 degrees warming. Nat. Clim. Change.

[27] Vogel MM (2017). Regional amplification of projected changes in extreme temperatures strongly controlled by soil moisture‐temperature feedbacks. Geophys. Res. Lett..

[28] Vautard R (2013). The simulation of European heat waves from an ensemble of regional climate models within the EURO-CORDEX project. Clim. Dyn..

[29] Stefanon M, D’Andrea F, Drobinski P (2012). Heatwave classification over Europe and the Mediterranean region. Environ. Res. Lett..

[30] Kenyon J, Hegerl GC (2008). Influence of modes of climate variability on global temperature extremes. J. Climate.

[31] Quesada B, Vautard R, Yiou P, Hirschi M, Seneviratne SI (2012). Asymmetric European summer heat predictability from wet and dry southern winters and springs. Nat. Clim. Change.

[32] Miralles DG, Teuling AJ, Van Heerwaarden CC, de Arellano JVG (2014). Mega-heatwave temperatures due to combined soil desiccation and atmospheric heat accumulation. Nat. Geosci..

[33] Perkins SE, Argüeso D, White CJ (2015). Relationships between climate variability, soil moisture, and Australian heatwaves. J. Geophys. Res.: Atmospheres.

[34] Loughran, T. F., Perkins‐Kirkpatrick, S. E. & Alexander, L. V. Understanding the spatio‐temporal influence of climate variability on Australian heatwaves. *Int. J. Climatol*. accepted (2016)

[35] Matthews TK, Wilby RL, Murphy C (2017). Communicating the deadly consequences of global warming for human heat stress. Proc. Nat. Acad. Sci..

[36] Kjellstrom T, Crowe J (2011). Climate change, workplace heat exposure, and occupational health and productivity in Central America. Int. J. Occ. Environ. Health.

[37] Bragazza L (2008). A climatic threshold triggers the die‐off of peat mosses during an extreme heat wave. Glob. Ch. Biol..

[38] Harris, R. M. B. *et al*. The certainty of unpredictability: Climate change, extreme events and biodiversity impacts. *Nat. Clim. Change*, in review (2017).

[39] Gent P (2011). The community climate system model version 4. J. Climate.

[40] Fischer EM, Beyerle U, Knutti R (2013). Robust spatially aggregated projections of climate extremes. Nat. Clim. Change.

[41] Giorgi F, Francisco R (2000). Uncertainties in regional climate change prediction: a regional analysis of ensemble simulations with the HADCM2 coupled AOGCM. Clim. Dyn..

[42] Hirsch AL, Pitman AJ, Kala J (2014). The role of land cover change in modulating the soil moisture‐temperature land‐atmosphere coupling strength over Australia. Geophys. Res. Lett..

[43] King AD (2015). The timing of anthropogenic emergence in simulated climate extremes. Environ. Res. Lett..

[44] Coumou D, Robinson A (2013). Historic and future increase in the global land area affected by monthly heat extremes. Environ. Res. Lett..

[45] Zander KK, Botzen WJ, Oppermann E, Kjellstrom T, Garnett ST (2015). Heat stress causes substantial labour productivity loss in Australia. Nat. Clim. Change.

[46] Smith KR (2016). The last Summer Olympics? Climate change, health, and work outdoors. The Lancet.

[47] Richardson M, Cowtan K, Hawkins E, Stolpe MB (2016). Reconciled climate response estimates from climate models and the energy budget of Earth. Nat. Clim. Change.

[48] Otto A (2013). Energy budget constraints on climate response. Nat. Geosci..

[49] Parker TJ, Berry GJ, Reeder MJ (2013). The influence of tropical cyclones on heat waves in Southeastern Australia. Geophys. Res. Lett..

[50] Mueller B, Seneviratne SI (2012). Hot days induced by precipitation deficits at the global scale. Proc. Nat. Acad. Sci..

[51] Perkins SE (2015). A review on the scientific understanding of heatwaves—their measurement, driving mechanisms, and changes at the global scale. Atmos. Res..

[52] Collins, M. *et al*. Long-term Climate Change: Projections, Commitments and Irreversibility. In: Climate Change 2013: The Physical Science Basis. Contribution of Working Group I to the Fifth Assessment Report of the Intergovernmental Panel on Climate Change [Stocker, T. F. *et al*. (eds)]. Cambridge University Press, Cambridge, United Kingdom and New York, NY, USA (2013).

[53] Peters GP (2013). The challenge to keep global warming below 2 C. Nat. Clim. Change.

