# Author Correction: Changes in regional heatwave characteristics as a function of increasing global temperature

**DOI:** 10.1038/s41598-018-23085-z

**Published:** 2018-03-12

**Authors:** S. E. Perkins-Kirkpatrick, P. B. Gibson

**Affiliations:** 1 Climate Change Research Centre, UNSW Australia, NSW, 2052 Australia; 2 ARC Centre of Excellence for Climate System Science, UNSW Australia, NSW, 2052 Australia

Correction to: *Scientific Reports* 10.1038/s41598-017-12520-2, published online 25 September 2017

This Article contains an error in the legend of Figure 4.

“Ensemble spread (99th percentile – 1st percentile) of increases in the number of heatwave projected by the CMIP5 ensemble (blue) and CESM ensemble (purple) per 0.5 °C global warming, for (a) Alaska; (b) the Mediterranean; (c) Australia; (d) East Asia; (e) the Amazon, and (f) East Africa. These regions were chosen as they are representative of all 21 regions analysed. Note that the spread due to internal variability (estimated from CESM) is reasonably consistent across the thresholds. See table S.2 for region boundaries”.

should read:

“Ensemble spread (99th percentile – 1st percentile) of increases in the number of heatwave days projected by the CMIP5 ensemble (blue) and CESM ensemble (purple) per 0.5 °C global warming, for (a) Alaska; (b) the Mediterranean; (c) Australia; (d) East Asia; (e) the Amazon, and (f) East Africa. These regions were chosen as they are representative of all 21 regions analysed. Note that the spread due to internal variability (estimated from CESM) is reasonably consistent across the thresholds. See table S.2 for region boundaries”.

Additionally, this Article contains an error in the title of Table 3.

“Median estimate of warming per °C global warming due to internal variability. (CESM(99th–1st)/CMIP5 (99th–1st))”.

should read:

“The fraction (expressed as a percentage) of change in each regional heatwave characteristic due to internal variability. 100*(CESM(99th-1st)/CMIP5 (99th-1st))”.

