# Supplementary material for: Changes in regional heatwave characteristics as a function of increasing global temperature
Source: Sci Rep. 2017 Sep 25;7:12256. doi: 10.1038/s41598-017-12520-2 (PMC5613001; doi:10.1038/s41598-017-12520-2)
Supplement: Supplementary file 1 — supplementary material [file 41598_2017_12520_MOESM1_ESM.pdf]

Supplementary material

Changes in regional heatwave characteristics as a function of increasing global temperature

S.E. Perkins-Kirkpatrick[1,2], P.B. Gibson[1,2]

1. Climate Change Research Centre, UNSW Australia, NSW, 2052, Australia
2. ARC Centre of Excellence for Climate System Science, UNSW Australia, NSW, 2052, Australia.

For submission to *Scientific Reports*

14th April, 2017

Corresponding author:

Dr Sarah E. Perkins-Kirkpatrick

ARC DECRA Research Fellow

Climate Change Research Centre & ARC Centre of Excellence for Climate System Science

UNSW Australia

NSW, 2052, Australia

Sarah.kirkpatrick@unsw.edu.au

Ph: +61293850367

Fax: +61293858969

Model	Origin	Realization used
ACCESS1-0	Australia	1
Bcc-csm1-1-m	China	1
CanESM2	Canada	1
CCSM4	USA	2
CESM1-BCG	USA	1
CESM1-CAM5	USA	1
CMCC-CESM	Italy	1
CMCC-CM	Italy	1
CMCC-CMS	Italy	1
CNRM-CM5	France	1
CSIRO-Mk3-6-0	Australia	4
FGOALS-s2	China	1
GFDL-CM3	USA	1
GFDL-ESM2G	USA	1
GFDL-ESM2M	USA	1
HadGEM2-CC	UK	1
HadGEM2-ES	UK	1
IPSL-CM5A-LR	France	2
ISPL-CM5A-MR	France	1
IPSL-CM5B-LR	France	1
MIROC5	Japan	1
MIROC-ESM-CHEM	Japan	1
MIROC-ESM	Japan	1
MPI-ESM-LR	Germany	2
MPI-ESM-MR	Germany	1
MRI-CGCM3	Germany	1
NorESM1-M	Norway	1

Table S.1 CMIP5 models employed, which have daily data across the historical (1861-2005) and RCP8.5 (2006-2100) experiments. The right column indicates which realization number was used to represent each model.

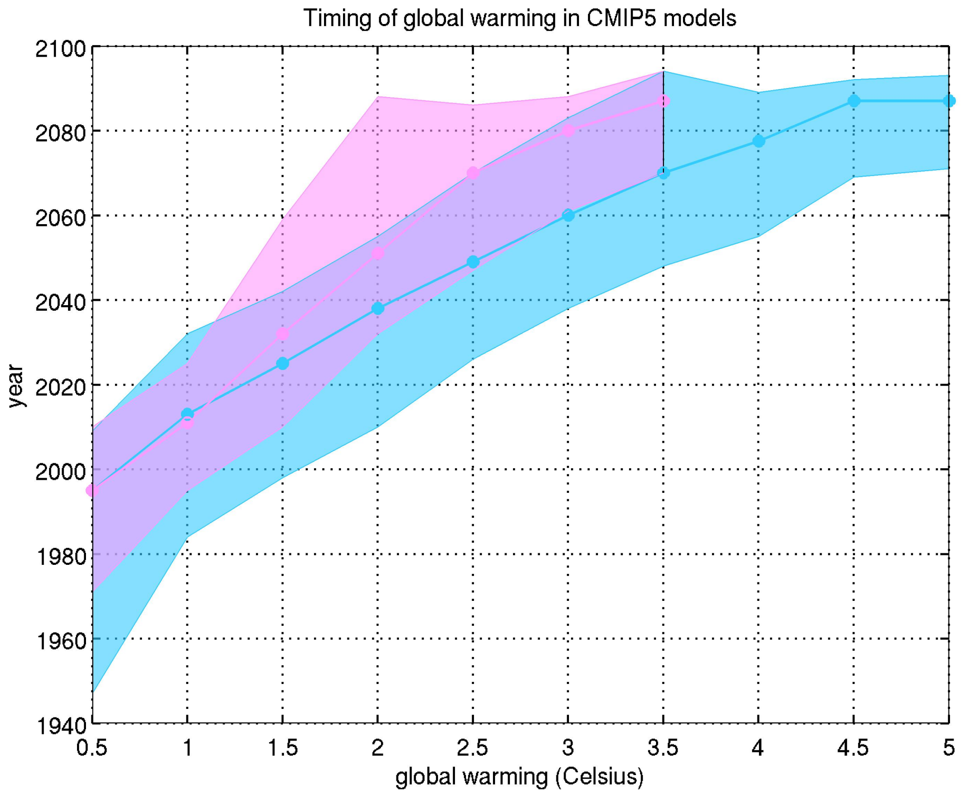

Figure S.1 – Timing of global warming in the CMIP5 models as a function of °C for RCP8.5 (blue) and RCP4.5 (purple). Note that prior to 2006 data is based on the historical experiment for both scenarios. Solid lines indicate the ensemble median, while shading is the respective 1st and 99th ensemble percentile for each experiment. Note that global warming thresholds of 1.5°C or warmer occur later in RCP4.5, with warming limited to 3.5°C by 2100. However, our analysis suggested very little difference between the relationship of global warming and regional changes in heatwaves. Such changes would simply occur later in RCP4.5 than RCP8.5. The same models and realizations were used across all experiments.

Acronym	Name	Latitude (°)	Longitude (°)
AUS	Australia	45S-11S	110E-155E
AMZ	Amazon Basin	20S-12N	82W-34W
SSA	Southern South America	56S-20S	75W-40W
CAM	Central America	10N-30N	116W-83W
WNA	Western North America	30N-60N	130W-103W
CNA	Central North America	30N-50N	103W-85W
ENA	Eastern North America	25N-50N	85W-60W
ALA	Alaska	60N-72N	170W-103W
GRL	Greenland	50N-85N	103W-10W
MED	Mediterranean Basin	30N-48N	10W-40E
NEU	Northern Europe	48N-75N	10W-40E
WAF	Western Africa	12S-18N	20W-22E
EAF	Eastern Africa	12S-18N	22E-52E

SAF	Southern Africa	35S-12S	10E-52E
SAH	Sahara	18N-30N	20W-65E
SEA	Southeast Asia	11S-12N	95E-155E
EAS	East Asia	20N-50N	100E-145E
SAS	South Asia	5N-30N	65E-100E
CAS	Central Asia	30N-50N	40E-75E
TIB	Tibet	30N-50N	75E-100E
NAS	North Asia	50N-70N	40E-180E

Table S.2 – region names and bounds used in the main text of this study, as taken from Giorgi and Francisco (2000).
